# The complete chloroplast genome of *Yunnanopilia longistaminea* (Opiliaceae), an endemic species in southwest China

**DOI:** 10.1080/23802359.2019.1677194

**Published:** 2019-10-16

**Authors:** Zeli Zhu, Chen Shi, Ninahui Cai, Xiaotong Ci, Jinyu Peng, Anan Duan, Dawei Wang

**Affiliations:** a Key Laboratory for Forest Resources Conservation and Utilization in the Southwest Mountains of China Ministry of Education, Southwest Forestry University, Kunming, China;; b Key Laboratory for Forest Genetic and Tree Improvement and Propagation in Universities of Yunnan Province, Southwest Forestry University, Kunming, China

**Keywords:** *Yunnanopilia longistaminea*, endemic species, chloroplast genome, phylogenetic analysis

## Abstract

The complete chloroplast genome sequence of *Yunnanopilia longistaminea*, an endemic species in southwest China, is presented in this study. The total genome size of *Y. longistaminea* was 148,503 bp in length, with a typical quadripartite structure including a pair of inverted repeat (IRs, 28,075 bp) regions separated by a large single copy (LSC, 84,547bp) region and a small single copy (SSC, 7805 bp) region. The all GC content was 37.3%. The genome contains 117 genes, including 72 protein-coding genes, 37 tRNA genes, and 8 rRNA genes, 13 genes contain a single intron, and 3 genes have two introns. Further, a maximum-likelihood (ML) phylogenetic tree results that *Y. longistaminea* was closely related to the genera of *Champereia manillana*.

*Yunnanopilia longistaminea* is a tropical evergreen woody plant belongs to the genus Opiliaceae in the family *Yunnanopilia* which mainly distributed in southwest China (Pu 2014). It is especially valued for nutrition and medicine because the leaves are rich in Vitamin C, iditol and potassium (Xu et al. 2014). It is well known as an edible vegetable by the local ethnic minorities. However, due to its Small reserves, narrow distribution (Yang 2013) and the excessive excavation of people, it is already facing the danger of extinction in the wild (Shi 2011).

Chloroplast genomes are widely used for phylogeny (Xue et al. 2012), DNA barcoding (Dong et al. 2014), genome evolution and species conservation (Dong et al. 2013). Up to now, The chloroplast genome of many species has been reported, such as *Champereia manillana* (Yang et al. 2017), *Zanthoxylurm bungeanum* (Liu and Wei 2017) and *Fabaceae* (Kato et al. 2000). In this study, we reported the complete chloroplast genome sequence of *Y. longistaminea* based on the Illumina pair-end sequencing data.

Leaf samples of *Y. longistaminea* were collected from Puer, Yunnan, China (geospatial coordinates: 101°49′35″E, 23°58′12″N; altitude: 1046 m). The Total DNA samples (YL 1-3) was extracted with Magnetic beads plant genomic DNA preps Kit (TSINGKE Biological Technology, Beijing, China) and kept at the Key Laboratory for Forest Genetic and Tree Improvement and Propagation in Universities of Yunnan Province, Southwest Forestry University, Kunming, China. Total DNA was used the Illumina Hiseq X platform to sequenced. Approximately 5.73 GB of raw data were genomic paired-end library of 150 bp length. Then, the raw data were used to assemble the complete Cp genome made by the software of GetOrganelle (Jin et al. 2018) with *Champereia manillana* as the reference. Genome annotation was performed with the programme Geneious R8 (Biomatters Ltd, Auckland, New Zealand), and then manually confirmed by comparison with the homologues *Champereia manillana*. The CpDNA sequence of *Y. longistaminea* was submitted to GenBank (accession number: MN444856).

The complete Cp genome of *Y. longistaminea* is 148,503 bp in size, which comprising a large single copy (LSC) region with a size of 84,547 bp, a small single copy (SSC) region of with a size of 7805 bp, separated by a pair of inverted repeats of 28,075 bp. The GC content of all *Y. longistaminea* Cp genome is 37.3%, while the GC contant of LSC was 35.2%, and the corresponding values of SSC and IR regions are 27.8% and 41.9%, respectively. A total of 117 function genes are contained in the chloroplast genome of *Y. longistaminea*, including 72 protein-coding genes, 37 tRNA genes, and 8 rRNA genes. 5 protein-coding genes, 8 tRNA genes, and all of rRNA genes were duplicated in IR regions. Among these function genes, 13 genes (rpoC1, rpl2, petB, rpl16, atpF, petD, rps16, trnK-UUU, trnI-GAU, trnA-UGC, trnG-UCC, trnV-UAC, trnL-UAA.) have a single intron and three genes (rps12, ycf3, and clpP) contain two introns.

To determine the phylogenetic location of *Y. longistaminea,* maximum-likelihood (ML) phylogenetic tree was reconstructed within the family of *Santalales* with fully sequenced chloroplast genomes, 16 complete chloroplast genome of *Santalales* were obtained from GenBank, the plastomes of *Viscum coloratum* in the family of *Santalales* were used as out-group. The chloroplast genome sequences of *Santalales* were aligned using MAFFT version 7 software (Katoh and Standley 2013) to reconstruct the phylogeny of the family of *Santalales*. The ML method for phylogenetic was reconstructed by using RA × ML version 8 programme with 1000 bootstrap replicates which based on TVM + F + I + G4 model (Stamatakis 2014). The phylogenetic tree revealed that *Y. longistaminea* was closely related to *Champereia manillana* (Figure 1). Moreover, the chloroplast genome of *Y. longistaminea* will provide useful information for phylogenetic studies and genomic resources available of Opiliaceae species.

**Figure. 1:**
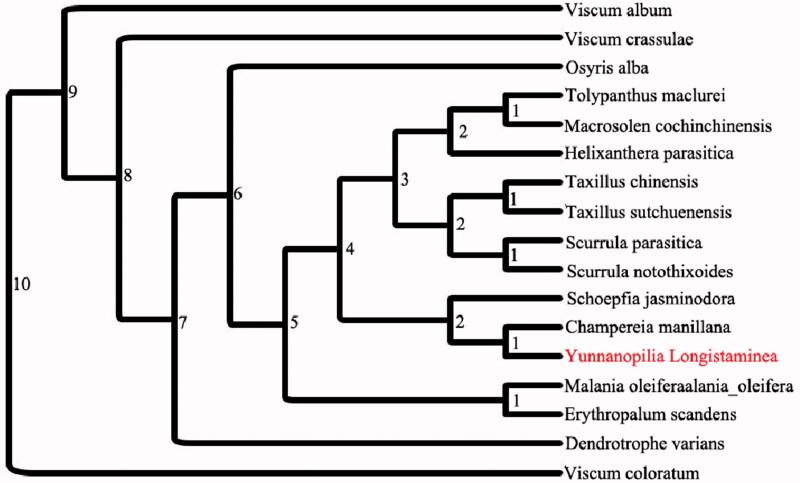
Phylogenetic relationships among 16 complete chloroplast genomes of *Santalales.* Bootstrap support values are given at the nodes. Chloroplast genome accession number used in this phylogeny analysis: *Viscum album*: KT003925.1; *Viscum crassulae*: KT070881.1; *Osyris alba*: KT070882.1; *Dendrotrophe varians*: MF592987.1; *Tolypanthus maclurei*: MH922027.1; *Malania oleifera:* NC_039426; *Schoepfia jasminodora*: NC_034228.1; *Champereia manillana*: NC-034931.1; *Viscum coloratum*: NC-035414.1; *Taxillus chinensis*: NC-036306.1; *Taxillus sutchuenensis*: NC_036307.1; *Erythropalum scandens*: NC_036759.1; *Helixanthera parasitica*: NC_039375.1; *Macrosolen cochinchinensis*: NC_039376.1; *Scurrula parasitica*: NC_040862.1; *Scurrula notothixoides*: NC_041305.1.
